# ECCM Schemes against Deception Jamming Using OFDM Radar with Low Global PAPR

**DOI:** 10.3390/s20072071

**Published:** 2020-04-07

**Authors:** Xinhai Wang, Gong Zhang, Xiangmin Wang, Qingqing Song, Fangqing Wen

**Affiliations:** 1 Nanjing Marine Radar Institute, Nanjing 211153, China; wangxinhai@nuaa.edu.cn (X.W.); xiangmin0727@126.com (X.W.); ssongqq@163.com (Q.S.); 2 Key Lab of Radar Imaging and Microware Photonics, Nanjing University of Aeronautics and Astronautics, Nanjing 211106, China; gzhang@nuaa.edu.cn; 3 National Demonstration Center for Experimental Electrical and Electronic Education, Yangtze University, Jingzhou 434023, China; 4 State Key Laboratory of Marine Resource Utilization in South China Sea, Hainan University, Haikou 570228, China

**Keywords:** orthogonal frequency division multiplexing (OFDM) radar, electronic counter-countermeasures (ECCM), deception jamming, global peak-to-average power ratio, alternating direction method multipliers (ADMM)

## Abstract

In this paper, a type of effective electronic counter-countermeasures (ECCM) technique for suppressing the high-power deception jamming using an orthogonal frequency division multiplexing (OFDM) radar is proposed. Concerning the velocity deception jamming, the initial phases of the pulses transmitted in a coherent processing interval (CPI) are designed to minimize the jamming power within a specific range, forming a notch around the jamming in the Doppler spectrum. For the purpose of suppressing the range deception jamming and the joint range-velocity deception jamming, the phase codes of the subcarriers belonging to the OFDM pulses are optimized to minimize the jamming power, distributing some specific bands in the range and the range-velocity domain, respectively. According to Parseval’s theorem, the phase encoding, acting as the coding manner of the OFDM subcarriers can ensure that the energy of each OFDM symbol stays the same. It is worth noticing that the phase codes of the OFDM subcarriers can influence the peak-to-average power ratio (PAPR). Thus, an optimization problem is formulated to optimize the phase codes of the subcarriers under the constraint of global PAPR, which can regulate the PAPRs of multiple OFDM symbols at the same time. The proposed problem is non-convex; therefore, it is a huge challenge to tackle. Then we present a method named by the phase-only alternating direction method multipliers (POADMM) to solve the aforementioned optimization problem. Some necessary simulation results are provided to demonstrate the effectiveness of the proposed radar signaling strategy

## 1. Introduction

The electronic countermeasures (ECM) have experienced a rapid development with the military radar over the past three decades [1,2]. The electronic countermeasures principally intend to mask useful information by creating false targets more attractive than the true ones to deceive the radar systems. Notably, the jammer based on the digital radio frequency memory (DRFM) can impose the proper scaling, delay, and Doppler frequency on the intercepted radar waveform, implementing its retransmission. As a result, the radar resources such as the abilities of detection and tracking are consumed severely and meaninglessly, which causes the conventional techniques of the radar signal processing to be invalid. In a word, a modern radar is required to detect targets in the midst of high-power jamming. It is worth noticing that, recently, the orthogonal frequency division multiplexing (OFDM) technique has been introduced to the array signal processing [3,4,5], and the joint radar and communication system [6,7]. Considering the demands of the military, the ECCM technology of the OFDM radar is investigated in this paper.

To generate a velocity deception jamming (VDJ), the repeat jammer modulates a false Doppler frequency on the radar waveform reproduced by the DRFM. The range deception jamming (RDJ), such as the range gate pull off (RGPO), and so on, usually takes the form of presenting false targets with a precalculated position to prevent the active sensors detecting the targets of interest. The self-defense repeat jammer can also generate the joint range—velocity deception jamming (JRVDJ) by modulating the time delay together with Doppler frequency and imitating the physical characteristic of the target by radar cross-section (RCS) modulation. The popular strategies of resisting the deception jamming can be divided into two categories—data processing and waveform design.

Recently, some data processing algorithms have been developed to restrain deceiving jamming. For instance, the probability density function of the output of the matched filter is investigated for target detection in the presence of deception jamming in [7]. In [8,9], the schemes based on the generalized likelihood ratio test (GLRT) are developed to distinguish between targets and the ECM signals. Additionally, in [10], a kind of adaptive detector is designed by resorting to the criteria of GLRT and Wald test in the presence of completely unknown jamming. However, with the huge development of the ECM technique, the deception jammer can produce false targets whose power is even able to overwhelm the true targets. Hence, the GLRT, which is usually based on data, might be useless in these cases. Additionally, the performance of data-based methods might depend on the degree of suppressing the jamming in both radar reception and the part of signal processing because the left jamming power is likely to be still greater than the level of target echoes. Therefore, the approaches based on data processing have some limitations.

The second category of methods resisting repeat jamming focuses on waveform design. Specifically, pulse diversity technology is one of the most significant foundations of the achievement of the waveform design, which can be employed to resist the VDJ, RDJ, and JRVDJ. Owing to the variation at the pulse repetition interval (PRI) level, the different signatures between the echo signal reflected by the true target of interest and the jamming signal might provide the possibility to discriminate among targets and jamming utilizing an appropriate signal processing. For example, in [11], the phase-perturbed linear frequency modulated (LFM) signal developing a modulated version of the chirp waveform or varying the chirp rate at each PRI is investigated to suppress the range jamming in the synthetic aperture radar (SAR). It’s essential idea of severely restricting the effectiveness of the deception jamming at the output of the matched filter mainly involves coding waveforms in such a fashion that the transmitted waveform at the current PRI is perpendicular to the jamming waveform made of the intercepted pulse at the previous PRI and retransmitted by the repeat jammer. A coupled sequential estimation algorithm to simultaneously obtain the range profiles of target and jamming is proposed in [12]. Each iteration achieves a set of linear filters for each range bin based on the minimization of the average output power. In [13], a train of multiple pulses exploiting the variations of the initial phases among the PRIs, optimized by the phase-only conjugate gradient method, is developed to count the velocity gate pull off. In addition, the optimal initial phases are designed in a multi-target scenario in [14]. The problem of resisting JRVDJ based on a single-input multiple-output (SIMO) radar system is discussed in [15]. The jamming and the true target are distinguished according to the Doppler diversity and spatial geometric correlation. In addition, a method based on the multiple-stage adaptive architecture is properly proposed to deal with the problem of target detection in the presence of noise-like and coherent interferers [16].

The orthogonal frequency division multiplexing (OFDM) technique is a vital technique that has been widely applied to the wireless communication systems since it can offer a high degree of spectrum efficiency and powerful ability to suppress the multi-path effect [17]. Not only is OFDM utilized for communication, but the OFDM signal has been paid much attention in radar applications. For instance, the combination of the OFDM technique and the SAR playing a critical role in remote sensing [18], marine, and terrestrial surveillance, together with the reconnaissance of enemy situation [19] has been investigated [20]. The multiple-input and multiple-output (MIMO) technique radar can be broadly applied to the direction-of-arrival (DOA) estimation [21,22,23,24] and wide-swath imaging [25] in a big way. Further, a newer system, so-called coprime array, becomes a key technology enabling a huge number of applications, including the DOA [26,27], and adaptive beamforming [28], and the orthogonality between the subcarriers of the OFDM signal can provide frequency diversity for the waveform of these array structures. By reason that the spectral components of an OFDM signal are orthogonal to each other, multiple packets of data can be sent simultaneously without mutual interference, each spectral component as a carrier transporting its corresponding data packet in time and frequency domains. Hence, the OFDM radar can provide the splendid characteristics of pulse-to-pulse diversity for the electronic counter-countermeasures (ECCM) [29]. Concretely, the pulsed initial phases and the modulated symbols of the OFDM subcarriers can vary rapidly at the PRI level easily. Nonetheless, the OFDM signal has a major drawback of a high peak-to-average power ratio (PAPR).

In this paper, we consider the ECCM schemes based on the OFDM radar against three types of deception jamming and the contributions of this paper are outlined as follows:

(1) We present the signal models based on a monostatic OFDM radar with the ability of pulse diversity in the presence of the different types of deception jamming. This work is utilized to pave the way to the ECCM scheme for improving the local signal-to-interference-and-noise ratio (SINR) via designing the proper waveform.
(2) A method of optimizing the initial phases is proposed to resist the VDJ. An optimization problem aiming at minimizing the energy near the false targets by devising the differences of the initial phases between the radar signal and the jamming signal repeated by jammer is formulated under a constant modulus constraint. Hence in order to force the components of the variable vector to be the same, we devise a method called phase-only BFGS (POBFGS) to obtain the phase vector of the optimization variable. It has some outstanding advantages over the existing methods in the performance of the algorithmic convergence.
(3) An ECCM scheme of optimizing the OFDM waveform is proposed to suppress the RDJ and the JRVDJ. In these two cases, two optimization problems are, respectively, formulated for the first time, and both of them comply with the criterion of minimizing the jamming energy near the false targets by designing the phase codes of the subcarriers (PCSs) of the OFDM pulses. Fortunately, they boil down to the same kind of non-convex optimization model. If the PCSs are changed, the PAPR may vary. The proper PAPR could help the transmitted signal to avoid suffering from severe nonlinear distortion. Therefore, a new concept of global PAPR is defined as the PAPR with regard to all OFDM pulses to be optimized, and controlling the global PAPR works as an indispensable constraint in the constructed mathematical model. Aiming at managing to get the optimal waveform, an algorithm called the phase-only alternating direction method of multipliers (POADMM) is devised to solve the formulated optimization problem in the phase domain.

The rest of the paper is organized as follows. Section 2 presents the signal models in the presence of various deception jamming. In Section 3, the optimization problems of the proposed ECCM schemes against three types of deception jamming are formulated. The novel methods of POBFGS and POADMM are proposed in Section 4. Section 5 provides various numerical simulations to validate the proposed methods. Ultimately, the conclusion is drawn in Section 6.

Notation: We adopt the notation of using boldface lowercase and uppercase letters for vectors and matrices, respectively. The notations of ${\mathbb{R}}^{N}$, ${\mathbb{C}}^{N}$, and ${\mathbb{C}}^{M\times N}$ are, respectively, the sets of *N*-dimensional vectors of real numbers, complex numbers, and *M* × *N* complex matrices. The operators ${\left(\cdot \right)}^{*}$ and ${\left(\cdot \right)}^{H}$, respectively, denote the conjugation and the conjugate transpose. The operator floor (.) is used to calculate the maximum integer less than the input entry. For any $x\in {\mathbb{C}}^{N}$, ‖x‖ means its Euclidian norm. In addition, arg{*x*} denotes the phase vector of *x*.|⋅|, ℜ{⋅} and ℑ{⋅} are the magnitude, real part, and image part of a complex-valued input. The operators ⊕ and ⊗ are the Hadamard operator and Kronecker product operator, respectively. Let ***I***, ***1***, and ***0*** denote the identity matrix, matrix of 1 and zero matrix (their size is determined from the context). The operators, Diag(⋅) and diag(⋅), are, respectively, the diagonal matrices formed by the entries of the vector argument and the column vector containing the principal diagonal of the matrix argument. ▽ denotes the gradient operator and vec(⋅) represents the vectorization of an input matrix.

## 2. Signal Model Under Deception Jamming

The velocity gate pull off (VGPO), RGPO, and the range-velocity gate pull off (RVGPO) are the classical types of deception jamming, respectively corresponding to VDJ, RDJ, and JRVDJ. A deception jammer initially produces a false target with the same parameter as the true target. However, the power of the false target is much higher than that of the true target for the purpose of enforcing the automatic radar gain to reduce its gain. Then the jammer modifies the parameters of the false target, dragging the false target from the location of the true target. At last, the jammer suddenly turns off, leaving the radar with a broken lock. Under some circumstances, there are even multiple targets produced simultaneously.

A traditional radar usually transmits a fixed signal which is easily detected and repeated by the hostile jammer based on DRFM. Hence, it often suffers from the deception jamming. In this paper, the OFDM radar with the ability of pulse diversity is supposed to enable the variation of the initial phase or the PCSs of the OFDM pulses. The reason why the encoding manner chooses the phase encoding is that the energy of each OFDM pulse can keep consistent according to the Parseval’s theorem. Thus, the level of difficulty in designing a radar transmitter can be degraded to a great extent.

Suppose that each target within the antenna beam can be regarded as a point scatter with constant RCS. The OFDM signal can be defined by

(1) $$s\left(t\right)=\frac{1}{N}\mathrm{rect}\left(\frac{t}{T_{p}}\right)\sum_{n=0}^{N-1}a_{m}\left(n\right)\exp\left(j2\pi f_{n}t\right)$$

where *N* is the number of the subcarriers, $f_{n}=n\Delta f$, n=0,1,⋯,N−1. Δf is the frequency space of the subcarriers. Let *T_p_* be the duration of the OFDM symbol and it is equal to the reciprocal of Δf. The vector $a_{m}={\left[e^{j\phi_{m,0}},e^{j\phi_{m,2}},\cdots ,e^{j\phi_{m,N-1}}\right]}^{T}$ represents the PCSs of the *m*-th OFDM pulse. 

**Figure 1:**
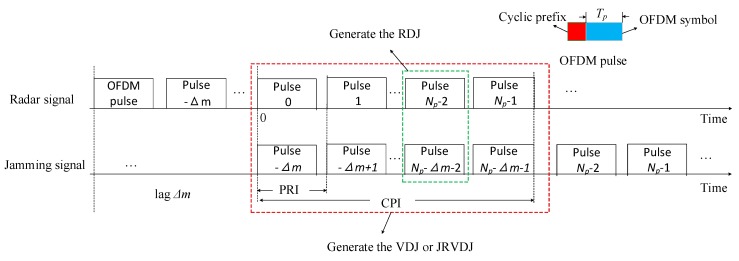
The mechanism of the deception jamming in a CPI with lag Δ*m*.

The repeat jammer based on the DRFM usually requires several PRIs to perceive the information of the radar parameters in the light of the captured radar signal and emits the processed pulses to generate multiple false targets to deceive the radar system. As shown in Figure 1, there are *N_p_* pulses during a coherent processing interval (CPI) and a complete OFDM pulse consists of an OFDM symbol and a cyclic prefix (CP). Assume that the pulse emitted by the jammer lags by Δm PRIs behind the pulse transmitted by the radar. In other words, at the *n*-th PRI, the jammer replicates the pulse that the radar transmitted at (n−Δm)-th PRI. At the (*N_p_* − 2)-th PRI, because of the existence of the (*N_p_* − Δ*m* − 2)-th pulse repeated by the jammer, an RDJ is formed. Furthermore, when the coherent processing is carried out in a CPI, the VDJ, or the JRVDJ could be generated.

### 2.1. Echo Signal Model in the Presence of VDJ

**Figure 2:**
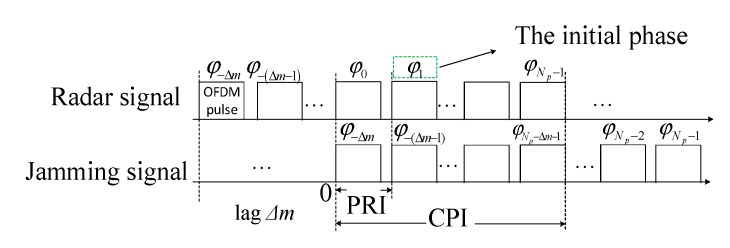
ECCM scheme of resisting the VDJ with lag Δ*m*.

Assume that there are *P* targets and *Q* false targets in the presence of the VDJ. The carrier frequency is denoted by $f_{c}$ and the pulse repetition interval (PRI) is represented by $T_{PRI}$. Let *c* denote the speed of light. For the VDJ, it is assumed that the different initial phases are imposed on the different OFDM pulses in a CPI. As can be seen in Figure 2 where the initial phase with regard to each pulse is marked, the timing relationship between the radar signal and the jamming signal is illustrated vividly, and the OFDM radar is capable of modulating the different initial phases on the different OFDM pulses. Then the echo signal of the *m*-th PRI can be expressed as

(2) $$s_{rv}(t)=\sum_{p=1}^{P}\sigma_{t,p}e^{j\varphi_{m}}s(t-\tau_{t,p})\exp(-j2\pi f_{c}\tau_{t,p})+\sum_{q=1}^{Q}\sigma_{j,q}e^{j\varphi_{m-\Delta m}}s(t-\tau_{j,q})\exp(-j2\pi f_{c}\tau_{j,q})$$

where $\tau_{t,p}$ and $\tau_{j,q}$ denote the delay of the *p*-th target and the delay of the *q*-th false target, respectively. The variables $\sigma_{t,p}$ and $\sigma_{j,q}$ represent the reflection coefficients of the *p*-th target and the *q*-th false target, respectively. Assuming that the initial range and velocity of the *p*-th target are, respectively, denoted by $R_{0,p}$ and $v_{t,p}$, the delay can be calculated by

(3) $$\tau_{t,p}=\frac{2\left[R_{0,p}-v_{t,p}(m-1)T_{PRI}\right]}{c}$$

where $\tau_{j,q}$ is determined by the jammer, and it would not influence the formation of the VDJ. Then $s_{rv}(t)$ can be rewritten as

(4) $$\begin{array}{l} s_{rv}(t)\\ =\sum_{p=1}^{P}{\tilde{\sigma }}_{t,p}e^{j\varphi_{m}}s(t-\tau_{t,p})\exp\left[j2\pi f_{dt,p}(m-1)\right]+\sum_{q=1}^{Q}{\tilde{\sigma }}_{j,q}e^{j\varphi_{m-\Delta m}}s(t-\tau_{j,q})\exp\left[j2\pi f_{dj,q}(m-1)\right] \end{array}$$

where

(5) $${\tilde{\sigma }}_{t,p}=\sigma_{t,p}\exp\left(-j4\pi \frac{R_{0,p}}{c}\right)$$

(6) $${\tilde{\sigma }}_{j,q}=\sigma_{j,q}\exp\left(-j4\pi \frac{R_{0,q}}{c}\right)$$

where $f_{dt,p}$ and $f_{dj,q}$ signify the normalized Doppler frequencies of the *p*-th target and the *q*-th false target, respectively. After eliminating the initial phase of the *m*-th pulse emitted by the radar, expression (4) can be modified into

(7) $$\begin{array}{ll} s{’}_{rv}(t) & =\sum_{p=1}^{P}{\tilde{\sigma }}_{t,p}s(t-\tau_{t,p})\exp\left[j2\pi f_{dt,p}(m-1)\right]\\ & +\sum_{q=1}^{Q}{\tilde{\sigma }}_{j,q}e^{j\left(\varphi_{m-\Delta m}-\varphi_{m}\right)}s(t-\tau_{j,q})\exp\left[j2\pi f_{dj,q}(m-1)\right] \end{array}$$

From expression (7), the second term related to the false targets possesses an extra phase $e^{j\left(\varphi_{m-\Delta m}-\varphi_{m}\right)}$. Hence, relying on the Doppler filter bank, the signal power of the true targets concentrates on the frequency points $f_{dt,p}$, *p* = 1, 2, …, *P*, whereas the power distribution of the jamming signal has something to do with $s_{v,\Delta \varphi ,\Delta m}\in {\mathbb{C}}^{N_{p}\times 1}$ which is defined by

(8) $$s_{v,\Delta \varphi ,\Delta m}={\left[e^{j\left(\varphi_{{}_{0}-\Delta m}-\varphi_{{}_{0}}\right)},e^{j\left(\varphi_{1-\Delta m}-\varphi_{1}\right)},\cdots ,e^{j\left(\varphi_{N_{p}-1-\Delta m}-\varphi_{N_{p}-1}\right)}\right]}^{T}$$

Regarding the *q*-th false target, the response of the Doppler filter bank at the frequency point *f* is

(9) $$s_{d}(f)=d_{q}{\left(f\right)}^{T}s_{v,\Delta \varphi ,\Delta m}$$

where

(10) $$d_{q}\left(f\right)={\left[\begin{array}{cccc} 1 & e^{j2\pi \left(f_{dj,q}-f\right)\cdot 1} & \cdots & e^{j2\pi \left(f_{dj,q}-f\right)\cdot \left(N_{p}-1\right)} \end{array}\right]}^{T}$$

### 2.2. Echo Signal Model in the Presence of RDJ

**Figure 3:**
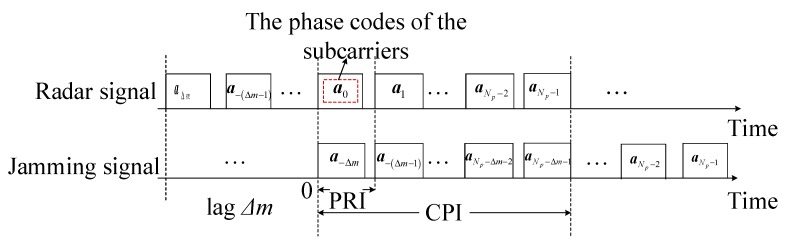
ECCM scheme of resisting the RDJ and JRVDJ with lag Δ*m*.

The RDJ usually forms several false targets in the fast time domain. As shown in Figure 3, the echo signal in the current PRI involves two parts: the reflected radar signal and the jamming signal, which can be expressed as

(11) $$s_{rr}\left(t\right)=\sum_{p=1}^{P}s_{r}{}_{t,p}\left(t\right)+\sum_{q=1}^{Q}s_{rj,q}\left(t\right)$$

where $s_{r}{}_{t,p}\left(t\right)$ and $s_{rj,q}\left(t\right)$ denote the echoes of the *p*-th target and the *q*-th false target, respectively. Concretely, they are given by

(12) $$s_{r}{}_{t,p}\left(t\right)=\frac{1}{N}\sum_{n=0}^{N-1}\sigma_{t,p}\mathrm{rect}\left(\frac{t-\tau_{t,p}}{T_{p}}\right)a_{m}\left(n\right)\exp\left[j2\pi f_{n}(t-\tau_{t,p})\right]\exp\left(-j2\pi f_{c}\tau_{t,p}\right)$$

(13) $$s_{rj,q}\left(t\right)=\frac{1}{N}\sum_{n=0}^{N-1}\sigma_{j,q}\mathrm{rect}\left(\frac{t-\tau_{j,q}}{T_{p}}\right)a_{m-\Delta m}\left(n\right)\exp\left[j2\pi f_{n}(t-\tau_{j,q})\right]\exp\left(-j2\pi f_{c}\tau_{j,q}\right)$$

As a result, their corresponding expression in the frequency domain is shown as follows:

(14) $$s_{ft,p}\left(f\right)=\frac{1}{N}\sum_{n=0}^{N-1}\sigma_{t,p}a_{m}\left(n\right)\exp\left[-j2\pi \left(f_{n}+f_{c}\right)\tau_{t,p}\right]\exp\left[j\pi \left(f_{n}-f\right)T_{p}\right]\mathrm{sinc}\left[j\pi \left(f_{n}-f\right)T_{p}\right]$$

(15) $$s_{fj,q}\left(f\right)=\frac{1}{N}\sum_{n=0}^{N-1}\sigma_{j,q}a_{m-\Delta m}\left(n\right)\exp\left[-j2\pi \left(f_{n}+f_{c}\right)\tau_{j,q}\right]\exp\left[j\pi \left(f_{n}-f\right)T_{p}\right]\mathrm{sinc}\left[j\pi \left(f_{n}-f\right)T_{p}\right]$$

After sampling the signal $s_{rr}\left(t\right)$ with the Nyquist frequency, the discrete versions of expressions (14) and (15) are, respectively, modified into:

(16) $$s_{ft,p}\left(k\right)=T_{p}\sigma_{t,p}a_{m}\left(k\right)\exp\left[-j2\pi \left(k+\frac{f_{c}}{\Delta f}\right)\tau {’}_{t,p}\right],\;\;\;k=0,1,\cdots ,N-1$$

(17) $$s_{fj,q}\left(k\right)=T_{p}\sigma_{j,q}a_{m-\Delta m}\left(k\right)\exp\left[-j2\pi \left(k+\frac{f_{c}}{\Delta f}\right)\tau {’}_{j,q}\right],\;\;\;k=0,1,\cdots ,N-1$$

where $\tau {’}_{t,p}$ and $\tau {’}_{j,q}$ are the normalized form of the $\tau_{t,p}$ and $\tau_{j,q}$ with respect to $T_{p}$. Then the PCSs should be decoded and the expressions (16) and (17), respectively, turn into:

(18) $${\tilde{s}}_{ft,p}\left(k\right)=T_{p}\sigma_{t,p}\exp\left[-j2\pi \left(k+\frac{f_{c}}{\Delta f}\right)\tau {’}_{t,p}\right]$$

(19) $${\tilde{s}}_{fj,q}\left(k\right)=T_{p}\sigma_{j}e^{j(\phi_{m-\Delta m,k}-\phi_{m,k})}\exp\left[-j2\pi \left(k+\frac{f_{c}}{\Delta f}\right)\tau_{j}’\right]$$

From the expression (19), the energy distribution of the false target is affected by the phase differences of the corresponding subcarriers possessed by the radar signal at the *m*-th PRI and the jamming signal. Hence, to highlight this point, the PCSs of each pulse are marked in Figure 3. At the 0-th PRI, ***a***_0_ and ***a***_−Δ*m*_ can influence the distribution of the jamming energy. Let the vector of the phase differences between the subcarriers of ***a***_0_ and ***a***_−Δ*m*_ be denoted by $s_{r,m,\Delta m}={\left[e^{j(\phi_{m-\Delta m,0}-\phi_{m,0})},e^{j(\phi_{m-\Delta m,2}-\phi_{m,2})},\cdots ,e^{j(\phi_{m-\Delta m,N-1}-\phi_{m,N-1})}\right]}^{T}$. To obtain the range of the target, the expression in the frequency domain is supposed to be recovered to the version in time domain. Then the radar signal and the jamming signal are respectively given by

(20) $$s_{rt,p}\left(t’\right)=\frac{T_{p}}{N}\sigma_{t,p}\sum_{k=0}^{N-1}\exp\left[j2\pi k\left(t’-\tau {’}_{t,p}\right)\right]\exp\left(-j2\pi \frac{f_{c}}{\Delta f}\tau {’}_{t,p}\right)$$

(21) $$s_{rj,q}\left(t’\right)=\frac{T_{p}}{N}\sigma_{j,q}\sum_{k=0}^{N-1}s_{r,m,\Delta m}\left(k\right)\cdot \exp\left[j2\pi k\left(t’-\tau {’}_{j,q}\right)\right]\exp\left(-j2\pi \frac{f_{c}}{\Delta f}\tau {’}_{j,q}\right)$$

The resulting expression of the complete signal can be put into

(22) $$\begin{array}{ll} {\hat{s}}_{r}\left(t’\right) & =\sum_{p=1}^{P}s_{rt,p}\left(t’\right)+\sum_{q=1}^{Q}s_{rj,q}\left(t’\right)\\ & =\sum_{p=1}^{P}\frac{T_{p}}{N}\sigma_{t,p}u_{t,p}^{T}v_{t’}+\sum_{q=1}^{Q}\frac{T_{p}}{N}\sigma_{j,q}{\left(s_{r,m,\Delta m}⊕u_{j,q}\right)}^{T}v_{t’} \end{array}$$

where

(23) $$u_{t,p}=e^{-j2\pi \frac{f_{c}}{\Delta f}\tau {’}_{t,p}}{\left[\begin{array}{cccc} 1 & e^{-j2\pi \tau {’}_{t,p}\cdot 1} & \cdots & e^{-j2\pi \tau {’}_{t,p}\cdot (N-1)} \end{array}\right]}^{T}$$

(24) $$u_{j,q}=e^{-j2\pi \frac{f_{c}}{\Delta f}\tau {’}_{j,q}}{\left[\begin{array}{cccc} 1 & e^{-j2\pi \tau {’}_{j,q}\cdot 1} & \cdots & e^{-j2\pi \tau {’}_{j,q}\cdot (N-1)} \end{array}\right]}^{T}$$

(25) $$v_{t’}={\left[\begin{array}{cccc} 1 & e^{j2\pi \cdot 1\cdot t’} & \cdots & e^{j2\pi \cdot (N-1)\cdot t’} \end{array}\right]}^{T}$$

Because of the existence of $s_{r,m,\Delta m}$, the jamming energy cannot be focused. For the *q*-th false target, the voltage amplitude of the jamming at the time t’ can be rewritten by:

(26) $$s_{rj,q}\left(t’\right)={\tilde{\sigma }}_{j,q}g_{q}^{T}s_{r,m,\Delta m}$$

where

(27) $${\tilde{\sigma }}_{j,q}=\frac{T_{p}}{N}\sigma_{j,q}$$

(28) $$g_{q}=v_{t’}⊕u_{j,q}$$

### 2.3. Echo Signal Model in the Presence of the Joint Range-Velocity Deception Jamming

In Figure 1, it is shown that the JRVDJ is formed by accumulating the pulses in a CPI. The echo signal involving the JRVDJ can be expressed as:

(29) $$s_{rrv}\left(m,t\right)=\sum_{p=1}^{P}s_{r}{}_{vt,p}\left(m,t\right)+\sum_{q=1}^{Q}s_{rvj,q}\left(m,t\right)$$

where $s_{r}{}_{vt,p}\left(m,t\right)$ and $s_{rvj,q}\left(m,t\right)$ denote the echo of the true target and the JRVDJ at the *m*-th PRI. They can be expressed by

(30) $$s_{rt,p}\left(m,t\right)=\frac{1}{N}\sum_{n=0}^{N-1}\sigma_{t,p}\mathrm{rect}\left(\frac{t-\tau_{t,p}}{T_{p}}\right)a_{m}\left(n\right)\exp\left[j2\pi f_{n}(t-\tau_{t,p})\right]\exp\left(j2\pi f_{dt,p}m\right)$$

(31) $$s_{rj,q}\left(m,t\right)=\frac{1}{N}\sum_{n=0}^{N-1}\sigma_{j,q}\mathrm{rect}\left(\frac{t-\tau_{j,q}}{T_{p}}\right)a_{m-\Delta m}\left(n\right)\exp\left[j2\pi f_{n}(t-\tau_{j,q})\right]\exp\left(-j2\pi f_{dj,q}m\right)$$

From the expression (31), the jammer based on DRFM adds the malicious delay $\tau_{j,q}$ and the Doppler frequency $f_{dj,q}$ normalized by the PRI, simultaneously. Decoding the subcarriers in the frequency domain, we convert the signal expression $s_{rrv}\left(m,t\right)$ into:

(32) $$\begin{array}{l} {\tilde{s}}_{rrv}\left(m,k\right)\\ =\sum_{p=1}^{P}T_{p}\sigma_{t,p}\exp\left(-j2\pi k\tau {’}_{t,p}\right)\exp\left[j2\pi f_{dt,p}m\right]+\sum_{q=1}^{Q}T_{p}\sigma_{j,q}e^{j(\phi_{m-\Delta m,k}-\phi_{m,k})}\exp\left(-j2\pi k\tau {’}_{j,q}\right)\exp\left[j2\pi f_{dj,q}m\right] \end{array}$$

where $\tau {’}_{t,p}$ is the normalized version of $\tau_{t,p}$ with respect to *T_p_*. In light of the expression (32), it can be observed that similar to the case of RDJ in expression (17), the phase differences of PCSs between the radar signal and the jamming signal may influence the jamming energy distribution in the Range–Doppler domain. According to Figure 3, the PCSs of a train of multiple pulses can be designed to regulate the jamming energy distribution. To obtain the Range-Doppler information of the target, the IDFT and DFT should be implemented in fast time *k* and the slow time *m*, respectively. Then, the expression (32) is transformed into:

(33) $${\hat{s}}_{rrv}\left(f_{d},t’\right)=\sum_{p=1}^{P}[\frac{T_{p}\sigma_{t,p}}{N}\sum_{k=1}^{N-1}\exp\left(-j2\pi k\tau {’}_{t,p}\right)\exp\left(j2\pi kt’\right)\cdot \sum_{m=0}^{N_{p}-1}\exp\left(j2\pi f_{dt,p}m\right)\exp\left(-j2\pi f_{d}m\right)]+{\hat{s}}_{rrvj,q}\left(f_{d},t’\right)$$

where

(34) $${\hat{s}}_{rrvj,q}\left(f_{d},t’\right)=\sum_{q=1}^{Q}\frac{T_{p}\sigma_{j,q}}{N}\sum_{k=0}^{N-1}\sum_{m=0}^{N_{p}-1}e^{j(\phi_{m-\Delta m,k}-\phi_{m,k})}\exp\left[j2\pi k\left(t’-\tau {’}_{j,q}\right)\right]\cdot \exp\left[j2\pi \left(f_{dj,q}-f_{d}\right)m\right]$$

For the *q*-th range-velocity deception jamming, the expression of the jamming signal corresponding to the second term of ${\hat{s}}_{rrv}\left(f_{d},t’\right)$ can be converted into the matrix form as follows:

(35) $$\begin{array}{l} {\hat{s}}_{rrvj,q}\left(f_{d},t’\right)\\ =\frac{T_{p}\sigma_{j,q}}{N}{\underset{g\left(f_{d}\right)}{\underset{︸}{\left[\begin{array}{c} 1\\ e^{j2\pi \left(f_{dj,q}-f_{d}\right)}\\ \vdots \\ e^{j2\pi \left(f_{dj,q}-f_{d}\right)\left(N_{p}-1\right)} \end{array}\right]}}}^{T}S_{\Delta \phi }\underset{h\left(t’\right)}{\underset{︸}{\left[\begin{array}{c} 1\\ e^{j2\pi \left(t’-\tau {’}_{j,q}\right)}\\ \vdots \\ e^{j2\pi \left(t’-\tau {’}_{j,q}\right)\left(N-1\right)} \end{array}\right]}}\\ =\frac{T_{p}\sigma_{j,q}}{N}h{\left(t’\right)}^{T}S_{\Delta \phi }^{T}g\left(f_{d}\right) \end{array}$$

where $S_{\Delta \phi }\in {\mathbb{C}}^{N_{p}\times N}$ is defined by

(36) $$\begin{array}{l} S_{\Delta \phi }\\ ={\left[a_{-\Delta m,0}a_{0,0}^{*},a_{-\Delta m+1,1}a_{1,1}^{*},\cdots ,a_{N_{p}-\Delta m,N-1}a_{N_{p},N-1}^{*}\right]}^{T}\\ =\left[\begin{array}{cccc} e^{j\left(\phi_{-\Delta m,0}-\phi_{0,0}\right)} & e^{j\left(\phi_{-\Delta m,1}-\phi_{0,1}\right)} & \cdots & e^{j\left(\phi_{-\Delta m,N-1}-\phi_{0,N-1}\right)}\\ e^{j\left(\phi_{-\Delta m+1,0}-\phi_{1,0}\right)} & e^{j\left(\phi_{-\Delta m+1,1}-\phi_{1,1}\right)} & \cdots & e^{j\left(\phi_{1-\Delta m,N-1}-\phi_{1,N-1}\right)}\\ \vdots & \vdots & \ddots & \vdots \\ e^{j\left(\phi_{N_{p}-1-\Delta m,0}-\phi_{N_{p}-1,0}\right)} & e^{j\left(\phi_{N_{p}-1-\Delta m,1}-\phi_{N_{p}-1,1}\right)} & \cdots & e^{j\left(\phi_{N_{p}-1-\Delta m,N-1}-\phi_{N_{p}-1,N-1}\right)} \end{array}\right] \end{array}$$

Furtherly, the expression (34) can be rewritten as:

(37) $${\hat{s}}_{rrvj,q}\left(f_{d},t’\right)=\frac{T_{p}\sigma_{j,q}}{N}\left[g^{T}\left(f_{d}\right)⊗h^{T}\left(t’\right)\right]\mathrm{vec}\left(S_{\Delta \phi }^{T}\right)$$

## 3. Proposed ECCM Schemes

For the above three types of deception jamming, the corresponding schemes are proposed in this section. The power of the jamming usually is much higher than that of the radar. For the jamming of VGPO, RGPO, and so on, the deceptive jamming can completely control the automatic gain control (AGC) of the radar and succeed in dragging the false targets from the location of the true targets to cover the real information. Assume that the false target is dragged within a certain range centered on the corresponding true target. In addition, the location of the target can be measured, resorting to the multi-channel processing schemes [13].

On account of the above facts, the ECCM schemes are proposed in this section. The main idea of all the schemes is that the energy distribution in a designated range around the false targets is minimized to improve the local signal-to-interference-plus-noise ratio (SINR).

### 3.1. Scheme of Suppressing VDJ

The power at the frequency point *f_d_* can be expressed by:

(38) $$p(f)=s_{v,\Delta \varphi ,\Delta m}^{H}E(f_{d})s_{v,\Delta \varphi ,\Delta m}$$

where $E(f_{d})=d^{*}(f_{d})d^{T}(f_{d})$ denotes the power density matrix. Further, the energy distributing in the *q*-th Doppler interval [*f_dl,q_*, *f_du,q_*] can be computed by:

(39) $$\int_{f_{dl,q}}^{f_{du,q}}s_{v,\Delta \varphi ,\Delta m}^{H}E(f_{d})s_{v,\Delta \varphi ,\Delta m}df_{d}=s_{v,\Delta \varphi ,\Delta m}^{H}\Gamma_{q}^{\left\{f_{dl,q},f_{du,q}\right\}}s_{v,\Delta \varphi ,\Delta m}$$

where the energy distribution matrix of the *q*-th Doppler interval is defined by:

(40) $$\Gamma_{q}^{\left\{f_{dl,q},f_{du,q}\right\}}\left(x,y\right)=\left\{ \begin{array}{cc} f_{du,q}-f_{dl,q}, & x=y\\ \frac{e^{j2\pi (f_{du,q}-f_{dj,q})(x-y)}-e^{j2\pi (f_{dl,q}-f_{dj,q})(x-y)}}{j2\pi (x-y)}, & x\neq y \end{array}\right.$$

According to both Figure 2 and the expression (38), the differences of the initial phases can be obtained by optimizing $s_{v,\Delta \varphi ,\Delta m}$ to minimize the jamming energy near the false targets. The optimization problem of suppressing the VDJ can be formulated by:

(41) $$\underset{\left|s_{v,\Delta \phi ,\Delta m}\left(n\right)\right|=1}{\min}s_{v,\Delta \varphi ,\Delta m}^{H}\sum_{q=1}^{Q}w_{q}\Gamma_{q}^{\left\{f_{dl},f_{du}\right\}}s_{v,\Delta \varphi ,\Delta m}$$

Once the initial phase of the first OFDM pulse is determined, the initial phases of pulses in the whole CPI are fixed. Moreover, the change of the initial phases would not influence the trait of the pulse PAPR. The problem (41) is a non-convex because of the existence of the constant modulus constraint, which is a challenging problem to deal with. The solution to the problem (41) is discussed in Section 4.

### 3.2. Scheme of Suppressing RDJ

As for the RDJ, the corresponding power at the normalized delay *t*’ can be calculated by:

(42) $$s_{rj,q}^{H}\left(t’\right)\cdot s_{rj,q}\left(t’\right)={\tilde{\sigma }}_{j,q}^{2}s_{r,m,\Delta m}^{H}g_{q}^{*}g_{q}^{T}s_{r,m,\Delta m}$$

Furtherly, the jamming energy within the time range [*t_1,q_*,*t_2,q_*] can be given by:

(43) $$\begin{array}{ll} \int_{t_{1,q}}^{t_{2,q}}s_{rj}^{H}\left(t’\right)\cdot s_{rj}\left(t’\right)dt’ & ={\tilde{\sigma }}_{j}^{2}\int_{t_{1,q}}^{t_{2,q}}s_{r,m,\Delta m}^{H}g_{q}^{*}g_{q}^{T}s_{r,m,\Delta m}dt’\\ & ={\tilde{\sigma }}_{j}^{2}s_{r,m,\Delta m}^{H}H^{\left\{t_{1,q},t_{2,q}\right\}}s_{r,m,\Delta m} \end{array}$$

Where

(44) $$H^{\left\{t_{1,q},t_{2,q}\right\}}\left(u,v\right)=\left\{ \begin{array}{cc} t_{2,q}-t_{1,q}, & u=v\\ \frac{e^{j2\pi \left(t_{2,q}-\tau_{j,q}\right)\left(v-u\right)}-e^{j2\pi \left(t_{1,q}-\tau_{j,q}\right)\left(v-u\right)}}{j2\pi (v-u)}, & u\neq v \end{array}\right.$$

For the purpose of suppressing the RDJ within some range intervals, the optimization problem of designing the phase differences between the PCSs of the current OFDM pulse and the intercepted one can be formulated by:

(45) $$\begin{array}{ll} \underset{s_{r,m,\Delta m},c_{-\Delta m}}{\min} & s_{r,m,\Delta m}^{H}\sum_{q=1}^{Q}w\left(q\right)H_{q}^{\left\{t_{1},t_{2}\right\}}s_{r,m,\Delta m}\\ s.t. & \left|s_{r,\Delta \phi ,\Delta m}\left(n\right)\right|=1\\ & \left|c_{-\Delta m}\left(n\right)\right|=1,n=1,2,\cdots ,N\\ & \frac{{\left\|A_{r}c_{-\Delta m}\right\|}_{\infty }^{2}}{\frac{1}{N_{p}N}{\left\|A_{r}c_{-\Delta m}\right\|}_{2}^{2}}=\gamma \end{array}$$

where $c_{-\Delta m}\left(n\right)$ denotes the PCSs belonging to the −Δ*m*–th OFDM pulse. The parameter γ is a preset constant for the global PAPR constraint and

(46) $$A_{r}=\left(I_{N_{c}}⊗M_{IDFT}\right)\left[\begin{array}{c} I_{N}\\ \mathrm{Diag}\left(s_{r,m,\Delta m}\right) \end{array}\right]$$

Let $M_{IDFT}\in {\mathbb{C}}^{N\times N}$ be the inverse Fourier transform matrix.

In the problem (45), the first constraint is used to force the modulus of the differences of the PCSs to be a constant. And the constant modulus constraint related to the PCSs of the intercepted −Δ*m*–th OFDM pulse is expressed by the second constraint. Since the variation of the PCSs caused the change of the pulse PAPR, the OFDM pulses are supposed to be optimized under the constraint of the global PAPR. The concept of global PAPR is defined as the PAPR calculated using all the concerned pulses. Accordingly, the third constraint in optimization problem (45) points out the global PAPR with regard to the designed pulses, resorting to the interworking of $s_{r,m,\Delta m}$, and $c_{-\Delta m}\left(n\right)$. Using $s_{r,m,\Delta m}$ and $c_{-\Delta m}\left(n\right)$, we can get the PCSs of all the OFDM pulses.

### 3.3. Scheme of Suppressing JRVDJ

In order to minimize the jamming energy, we are supposed to compute the power value in the Range–Doppler domain. Through expression (37), the power at the point (*t’*, *f_d_*) can be calculated by:

(47) $$\begin{array}{l} p_{rrrvj,q}\left(t’,f_{d}\right)={\left(\frac{T_{p}\left|\sigma_{j,q}\right|}{N}\right)}^{2}s_{rv,\Delta \phi }^{H}{\left(g^{T}\left(f_{d}\right)⊗h^{T}\left(t’\right)\right)}^{H},\\ \left(g^{T}\left(f_{d}\right)⊗h^{T}\left(t’\right)\right)s_{rv,\Delta \phi }={\left(\frac{T_{p}\left|\sigma_{j,q}\right|}{N}\right)}^{2}s_{rv,\Delta \phi }^{H}\left[g^{*}\left(f_{d}\right)g^{T}\left(f_{d}\right)⊗h^{*}\left(t’\right)h^{T}\left(t’\right)\right]s_{rv,\Delta \phi }. \end{array}$$

With regard to the *q*-th JRVDJ, its energy occupying the Delay-Doppler domain $t{’}_{q}\in [t_{1,q},t_{2,q}]$, $f_{d,q}\in [f_{dl,q},f_{du,q}]$ can be obtained by:

(48) $$\begin{array}{ll} E_{\left\{f_{dl,q},f_{du,q}\right\}}^{\left\{t_{1,q},t_{2,q}\right\}} & ={\left(\frac{T_{p}\sigma_{j,q}}{N}\right)}^{2}s_{rv,\Delta \phi }^{H}U_{q}^{\left\{t_{1,q},t_{2,q},f_{dl,q},f_{du,q}\right\}}s_{rv,\Delta \phi }\\ & ={\left(\frac{T_{p}\sigma_{j,q}}{N}\right)}^{2}s_{rv,\Delta \phi }^{H}\left(\Gamma_{q}^{\left\{f_{dl,q},f_{du,q}\right\}}⊗H_{q}^{\left\{t_{1,q},t_{2,q}\right\}}\right)s_{rv,\Delta \phi } \end{array}$$

where $s_{rv,\Delta \phi }=\mathrm{vec}\left(S_{\Delta \phi }^{T}\right)$ and

(49) $$U_{q}^{\left\{t_{1,q},t_{2,q},f_{dl,q},f_{du,q}\right\}}=\int_{f_{dl}}^{f_{du}}g^{*}\left(f_{d,q}\right)g^{T}\left(f_{d,q}\right)df_{d}⊗\int_{t_{1}}^{t_{2}}h^{*}\left(t{’}_{q}\right)h^{T}\left(t{’}_{q}\right)dt’\;\Gamma_{q}^{\left\{f_{dl,q},f_{du,q}\right\}}\left(u,v\right)=\left\{ \begin{array}{cc} f_{du,q}-f_{dl,q}, & \;u=v\\ \frac{e^{j2\pi (f_{du,q}-f_{dj,q})(u-v)}-e^{j2\pi (f_{dl,q}-f_{dj,q})(u-v)}}{j2\pi (u-v)}, & u\neq v \end{array}\right.\;H_{q}^{\left\{t_{1,q},t_{2,q}\right\}}\left(u,v\right)=\left\{ \begin{array}{cc} t_{2,q}-t_{1,q}, & u=v\\ \frac{e^{j2\pi \left(t_{2,q}-\tau_{j,q}\right)\left(v-u\right)}-e^{j2\pi \left(t_{1,q}-\tau_{j,q}\right)\left(v-u\right)}}{j2\pi (v-u)}, & u\neq v \end{array}\right.$$

For convenience, we assume $N_{p}=l\cdot \Delta m$ and *l* is a proper integer. For the purpose of suppressing the JRVDJ, the problem of minimizing the jamming energy in some Range–Doppler areas around the jamming locations is formulated as:

(50) $$\begin{array}{ll} \underset{s_{rv,\Delta \phi },c}{\min} & s_{rv,\Delta \phi }^{H}\left(\sum_{q=1}^{Q}w_{q}\Lambda_{q}^{\left\{t_{1,q},t_{2,q},f_{dl,q},f_{du,q}\right\}}\right)s_{rv,\Delta \phi }\\ s.t. & \left|s_{rv,\Delta \phi }\left(m\right)\right|=1,m=1,2,\cdots ,N_{p}N\\ & \left|c\left(n\right)\right|=1,n=1,2,\cdots ,\Delta mN\\ & \frac{{\left\|A_{rv}c\right\|}_{\infty }^{2}}{\frac{1}{N_{p}N}{\left\|A_{rv}c\right\|}_{2}^{2}}=\gamma \end{array}$$

where the vector c is formed by stacking the PCSs of the (Δ*m*~−1)-th OFDM pulses into a column vector in sequence and

(51) $$\Lambda_{q}^{\left\{t_{1,q},t_{2,q},f_{dl,q},f_{du,q}\right\}}=\Gamma_{q}^{\left\{f_{dl,q},f_{du,q}\right\}}⊗H_{q}^{\left\{t_{1,q},t_{2,q}\right\}}$$

(52) $$A_{rv,z}=\left(I_{N_{z}+1}⊗M_{IDFT}\right)\left[\begin{array}{c} I_{N}\\ \mathrm{Diag}\left(s_{rv\Delta ,0}\right)\\ \mathrm{Diag}\left(s_{rv\Delta ,1}\right)\\ \vdots \\ \mathrm{Diag}\left(s_{rv\Delta ,N_{z}}\right) \end{array}\right]$$

(53) $$\begin{array}{l} s_{rv\Delta }=s_{rv,\Delta \phi }⊙\exp\left[jT\arg\left(s_{rv,\Delta \phi }\right)\right]⊙\exp\left[jT^{2}\arg\left(s_{rv,\Delta \phi }\right)\right]⊙\cdots \\ ⊙\exp\left[jT^{N_{p}-2}\arg\left(s_{rv,\Delta \phi }\right)\right]⊙\exp\left[jT^{N_{p}-1}\arg\left(s_{rv,\Delta \phi }\right)\right] \end{array}$$

(54) $$T={\left(\begin{array}{ccccc} 0_{N\times \Delta m} & 0_{N\times \Delta m} & 0_{N\times \Delta m} & \cdots & 0_{N\times \Delta m}\\ I_{N\times \Delta m} & 0_{N\times \Delta m} & 0_{N\times \Delta m} & \ddots & \begin{array}{c} 0_{N\times \Delta m} \end{array}\\ 0_{N\times \Delta m} & I_{N\times \Delta m} & \ddots & \ddots & \begin{array}{c} 0_{N\times \Delta m} \end{array}\\ \vdots & & \ddots & 0_{N\times \Delta m} & 0_{N\times \Delta m}\\ \begin{array}{c} 0_{N\times \Delta m} \end{array} & \begin{array}{c} 0_{N\times \Delta m} \end{array} & \begin{array}{c} 0_{N\times \Delta m} \end{array} & \begin{array}{c} \begin{array}{c} I_{N\times \Delta m} \end{array} \end{array} & 0_{N\times \Delta m} \end{array}\right)}_{N_{p}N\times N_{p}N}$$

where $s_{rv\Delta }$ can be divided into l blocks, and $s_{rv\Delta ,n}$ represents the *n*-th block. Additionally, because the PCSs of the (−Δm~−1)-th pulses also need to be optimized, the number of the total pulses requiring to be designed is *N_c_* = *N_P_* + Δ*m*. The construction of the problem (50) resembles that of problem (45).

**Remark** **1.** *As for the scheme of suppressing the VDJ, the designed initial phases of the OFDM pulses in a CPI will not influence the global PAPR, and the initial phase of the first pulse can be selected according to the local conditions since it has nothing to do with the implementation of ECCM scheme. However, for the schemes of suppressing the RDJ and the JRVDJ, the subcarriers’ phase differences between the pulses and the coexisting jamming pulses are designed adaptively. Once the PCSs, including* $a_{-\Delta m}$ *, …,* $a_{-\Delta m+1}$ *in Figure 3 are fixed, the PCSs of all the pulses in a CPI can be determined, combining the subcarriers’ phase differences. It is worth noticing that the similarity between these two cases is that the signal PAPR may be influenced by the variations of the PCSs. Hence, the PCSs of the OFDM pulses intercepted initially, and the vector of the subcarriers’ phase differences should be jointly optimized to ascertain the optimal PCSs of all the OFDM pulses. For convenience,* Δm *is assumed to be equal to 1. Considering that the signal PAPR determines the working point of the power amplifier in the radar transmitter, the problems of suppressing RDJ and JRVDJ can be concluded in the same way as follows:*

(55) $$\begin{array}{ll} \underset{s,c}{\min} & s^{H}\Xi s\\ s.t. & \left|s\left(n\right)\right|=1,n=1,2,\cdots ,N_{p}N\\ & \left|c\left(m\right)\right|=1,m=1,2,\cdots ,N\\ & \frac{{\left\|Ac\right\|}_{\infty }^{2}}{\frac{1}{N_{p}N}{\left\|Ac\right\|}_{2}^{2}}=\gamma \\ & x=Ac \end{array}$$

*where*

(56) $$A=\left(I_{N_{c}}⊗M_{IDFT}\right)\left[\begin{array}{c} I_{N}\\ \mathrm{Diag}\left(s_{\Delta ,1}\right)\\ \mathrm{Diag}\left(s_{\Delta ,2}\right)\\ \vdots \\ \mathrm{Diag}\left(s_{\Delta ,N_{p}}\right) \end{array}\right]$$

(57) $$s_{\Delta }=s⊙\exp\left[jT\arg\left(s\right)\right]⊙\exp\left[jT^{2}\arg\left(s\right)\right]⊙\cdots ⊙\exp\left[jT^{N_{p}-2}\arg\left(s\right)\right]⊙\exp\left[jT^{N_{p}-1}\arg\left(s\right)\right]$$

*The vector* s *amounts to* $s_{r,\Delta \phi ,\Delta m}$ *or* $s_{rv,\Delta \phi }$ *and* c(n) *amounts to* $c_{-\Delta m}$ *. The vector* $s_{\Delta }$ *can be divided into* $N_{p}$ *blocks and* $s_{\Delta ,n}$ *represents the n-th block.*

## 4. Designing OFDM Waveform for ECCM

In this section, the approaches to solve the problems formulated in Section 3 are explained in detail.

### 4.1. Waveform Design for Suppressing the VDJ

For the purpose of suppressing the VDJ, the initial phases of OFDM pulses are optimized in expression (41). Although the optimization problem (41) is non-convex, it can be solved by some existing approaches, such as POVMM [30] and the method called phase-only conjugate gradient (POCG) [13]. Analogously, a method based on BFGS is utilized to find the phase vector of the optimal waveform vector in this paper, which is so-called the POBFGS as a matter of convenience. Assume that the phase vector with regard to $s_{v,\Delta \varphi ,\Delta m}$ is $\xi_{s}$. Based on [31], the derivative of the objective function in the optimization problem (41) with regard to $\xi_{s}$ is given as:

(58) $$\nabla_{\xi_{s}}\varpi \left(s_{v,\Delta \varphi ,\Delta m}\right)=ℑ\left\{diag\left(\Xi s_{v,\Delta \varphi ,\Delta m}s_{v,\Delta \varphi ,\Delta m}^{H}-s_{v,\Delta \varphi ,\Delta m}s_{v,\Delta \varphi ,\Delta m}^{H}\Xi \right)\right\}$$

Then the algorithm of POBFGS is concluded in detail in Algorithm 1.
**Algorithm 1:** Solving the problem (40) with POBFGS**Initialization:** Initialize the iteration number *l* = 0. And generate $\xi_{s,l}$ randomly and the stopping threshold value is set to be *ε*. Let ***H_0_*** = ***I****_Np_*. (The letter *l* in the subscript denotes the number of the iteration and $s_{v,l}$ is the value of $s_{v,\Delta \varphi ,\Delta m}$ at *l*-th iteration.) **For**
*l* = 0,1,2,3,… Calculate $\kappa (\xi_{s,l})$ and $g_{v,l}=\nabla_{\Phi }\kappa \left(\xi_{s,l}\right)$ using the expression (56). If $\left\|g_{l}\right\|\leq \varepsilon$, perform Step 4 and stop the algorithm; otherwise, perform Step 2.Find the searching step length $\alpha_{l}$ along $d_{l}=-H_{l}g_{l}$ and calculate the new point $\eta_{s,l+1}=\eta_{s,l}+\alpha_{l}d_{l}$. Update $s_{v,l+1}$, $g_{l+1}$.Calculate
$$y_{l}=g_{v,l+1}-g_{v,l},$$
$$v_{l}={\left(y_{l}^{T}Hy\right)}^{1/2}(\frac{s_{v,l}}{s_{v,l}^{T}y_{l}}-\frac{H_{l}y_{l}}{y_{l}^{T}H_{l}y_{l}}),$$
$$H_{l+1}=H_{l}+\frac{s_{v,l}^{T}s_{v,l}}{s_{v,l}^{T}y_{l}}-\frac{H_{l}y_{l}y_{l}^{T}H_{l}}{y_{l}^{T}H_{l}y_{l}}+v_{l}v_{l}.$$Continue Step 1 and *l* = *l* + 1.Obtain the optimal solution by $s_{v,\Delta \phi ,\Delta m}^{opt}=\exp(j\xi_{s,l})$.


### 4.2. Waveform Design for Suppressing the RDJ and JRVDJ

From the Section 3.2 and Section 3.3, the PCSs are designed to subdue the RDJ and JRVDJ. In these cases, the PAPR of the OFDM signal might vary, even deteriorate. Therefore, the PAPR constraint must be considered. And it is worth noticing that the PCSs of two OFDM pulses are designed to resist the RDJ, whereas the PCSs of all pulses in a CPI are optimized simultaneously to minimize the jamming energy around the locations of the false targets.

Since the optimization problem is handled in the phase domain, an algorithm called POADMM is proposed to tackle the problem (55). The optimal solution is obtained iteratively. In case that the constant modulus constraint is omitted, the argument Lagrange function corresponding to the problem (55) can be formed by:

(59) $${ℒ}_{\rho }\left(s,c,x,\lambda \right)=s^{H}\Xi s+ℜ\left\{\lambda^{H}\left(Ac-x\right)\right\}+\frac{\rho }{2}{\left\|Ac-x\right\|}_{2}^{2}$$

where its equivalent scaled form can be expressed by [32]

(60) $${ℒ}_{s,\rho }\left(s,c,x,\lambda \right)=s^{H}\Xi s+\frac{\rho }{2}{\left\|Ac-x+\frac{\lambda }{\rho }\right\|}_{2}^{2}$$

The framework of the proposed POADMM consists of the iterations as follows:

(61) $$s_{k+1}=\underset{s\in ℬ}{\arg\min}{ℒ}_{\rho }\left(s,c_{k},x_{k},\lambda_{k}\right)$$

(62) $$c_{k+1}=\underset{c\in C}{\arg\min}{ℒ}_{\rho }\left(s_{k+1},c,x_{k},\lambda_{k}\right)$$

(63) $$x_{k+1}=\underset{x\in D}{\arg\min}{ℒ}_{\rho }\left(s_{k+1},c_{k+1},x,\lambda_{k}\right)$$

(64) $$\lambda_{k+1}=\lambda_{k}+\rho \left(A_{k+1}c_{k+1}-x_{k+1}\right)$$

where

(65) $$ℬ=\left\{s|\left|s\left(m\right)\right|=1,m=1,2,\cdots ,N_{p}N\right\},\;C=\left\{c|\left|c\left(n\right)\right|=1,n=1,2,\cdots ,N\right\}$$

(66) $$D=\left\{x|\frac{\|x{\|}_{\infty }^{2}}{\frac{1}{N_{c}N}\|x\|}=\gamma \right\}$$

The subscripts of the primal variables $s_{k}$, $c_{k}$, $x_{k}$ and the dual variable $\lambda_{k}$, respectively denote their iteration counters. In the following, we develop a concrete solution to the above problems (61)–(64).

(1) Updating $s_{k+1}$

The subproblem (61) of updating s is expressed as:

(67) $$\begin{array}{l} \underset{s}{\min}\;{ℒ}_{\rho }\left(s,c_{k},x_{k},\lambda_{k}\right)\\ \mathrm{subject}\;\mathrm{to}\;\left|s\left(m\right)\right|=1,m=1,2,\cdots ,N_{p}N \end{array}$$

Owing to that the matrix ***A*** in (56) is related to the vector ***s***, the expression (61) is supposed to be converted to:

(68) $${ℒ}_{\rho }\left(s,c,x,\lambda \right)=s^{H}\Xi s+ℜ\left\{\lambda^{H}\left(B\left[\begin{array}{l} 1_{N}\\ s_{\Delta } \end{array}\right]-x\right)\right\}+\frac{\rho }{2}{\left\|B\left[\begin{array}{l} 1_{N}\\ s_{\Delta } \end{array}\right]-x\right\|}_{2}^{2}$$

where

(69) $$B=\left(I_{N_{c}}⊗M_{IDFT}\right)\left(I_{N_{c}}⊗\mathrm{Diag}\left(c\right)\right)$$

and the corresponding scaled form of the expression (68) can be given by:

(70) $${ℒ}_{s,\rho }\left(s,c,x,\lambda \right)=s^{H}\Xi s+\frac{\rho }{2}{\left\|B\left[\begin{array}{l} 1_{N}\\ s_{\Delta } \end{array}\right]-x+\frac{\lambda }{\rho }\right\|}_{2}^{2}$$

Thus, the subproblem (67) is equivalent to:

(71) $$\begin{array}{l} \underset{s}{\min}s^{H}\Xi s+\frac{\rho }{2}{\left\|B_{k}\left[\begin{array}{l} 1_{N}\\ s_{\Delta } \end{array}\right]-x_{k}+\frac{\lambda_{k}}{\rho }\right\|}_{2}^{2}\\ \mathrm{subject}\;\mathrm{to}\;\left|s\left(m\right)\right|=1,m=1,2,\cdots ,N_{p}N \end{array}$$

where the symbol $B_{k}$ corresponds to the value of the matrix B at the *k*-th iteration. Then the objective function in the problem (71) can be organized as:

(72) $${ℱ}_{s}\left(s\right)=s^{H}\Xi s+\frac{\rho }{2}\left(s_{\Delta }^{H}B_{k,\Delta }^{H}p+p^{H}B_{k,\Delta }s_{\Delta }\right)+\frac{\rho }{2}\left(N_{p}+{\left\|p\right\|}^{2}\right)$$

The detailed derivation is given in Appendix A.

Assuming that the phase vector with regard to s is denoted by $\eta_{s}$, the gradient vector $\nabla_{\eta_{s}}g\left(s\right)$ can be obtained in light of [31]. Therefore, the gradient of ${ℱ}_{s}\left(s\right)$ with respect to $\eta_{s}$ can be calculated by:

(73) $$\nabla_{\eta_{s}}{ℱ}_{s}\left(s\right)=\nabla_{\eta_{s}}g\left(s\right)+\frac{\rho }{2}\nabla_{\eta_{s}}r\left(s_{\Delta }\right)$$

where q is defined in Appendix A and

(74) $$\nabla_{\eta_{s}}g\left(s\right)=ℑ\left\{diag\left(\Xi ss^{H}-ss^{H}\Xi \right)\right\}$$

(75) $$\nabla_{\eta_{s}}r\left(s_{\Delta }\right)=-2ℑ\left(\begin{array}{c} \begin{array}{c} q_{\Delta ,1}^{T}{\tilde{s}}_{\Delta ,1}\\ q_{\Delta ,2}^{T}{\tilde{s}}_{\Delta ,2}\\ \vdots \end{array}\\ q_{\Delta ,i}^{T}{\tilde{s}}_{\Delta ,i}\\ \vdots \\ q_{\Delta ,N}^{T}{\tilde{s}}_{\Delta ,N} \end{array}\right)$$

(76) $${\tilde{s}}_{\Delta ,i}=\left[\begin{array}{c} s_{\Delta }\left(i\right)\\ s_{\Delta }\left(i+N\right)\\ \vdots \\ \begin{array}{c} s_{\Delta }\left(i+kN\right)\\ \begin{array}{c} \vdots \\ s_{\Delta }\left(i+\left[N_{p}-\mathrm{floor}\left(\frac{i}{N}\right)-1\right]N\right) \end{array} \end{array} \end{array}\right]$$

(77) $$q_{\Delta ,i}=\left[\begin{array}{c} q(i)\\ q(i+N)\\ \vdots \\ q(i+kN)\\ \vdots \\ q\left(i+\left[N_{p}-\;\mathrm{floor}\;\left(\frac{i}{N}\right)-1\right]N\right) \end{array}\right]$$

(2) Updating $c_{k+1}$

The PCSs of the first intercepted OFDM symbol can be obtained by solving the following problem:

(78) $$\begin{array}{ll} \underset{c}{\min} & {ℒ}_{\rho }\left(s_{k+1},c,x_{k},\lambda_{k}\right)\\ \mathrm{subject}\;\mathrm{to}\; & \left|c\left(n\right)\right|=1,n=1,2,\cdots ,N \end{array}$$

According to expression (60), the term $s^{H}\Xi s$ and c are irrelevant. In consequence, the optimization problem (78) is equivalent to:

(79) $$\begin{array}{ll} \underset{c}{\min} & {\left\|A_{k+1}c-x_{k}+\frac{\lambda_{k}}{\rho }\right\|}_{2}^{2}\\ \mathrm{subject}\;\mathrm{to}\; & \left|c\left(n\right)\right|=1,n=1,2,\cdots ,N. \end{array}$$

Owing to the existence of the non-convex constraint, the above optimization problem is NP-hard. Like the procedure of updating $s_{k+1}$, the approach of POBFGS can be utilized to solve the above problem.

Supposing that the objective function of the optimization problem (79) is denoted by ${ℱ}_{c}\left(c\right)$, the gradient of ${ℱ}_{c}\left(c\right)$ with respect to $\eta_{c}$, representing the phase vector of c can be given by:

(80) $$\nabla_{\eta_{c}}{ℱ}_{c}\left(c\right)=\nabla_{\eta_{c}}\kappa \left(c\right)+2ℑ\left\{{\left[{\left(x_{k}-\frac{\lambda_{k}}{\rho }\right)}^{H}A_{k+1}\right]}^{T}⊙c\right\}$$

where

(81) $$\nabla_{\eta_{c}}\kappa \left(c\right)=ℑ\left\{diag\left(\Lambda cc^{H}-cc^{H}\Lambda \right)\right\}$$

The detailed derivation is given in Appendix B.

(3) Updating $x_{k+1}$

The subproblem (63) with regard to x can be expressed by:

(82) $$\begin{array}{ll} \underset{x}{\min} & {\left\|A_{k+1}c_{k+1}-x+\frac{\lambda_{k}}{\rho }\right\|}_{2}^{2}\\ \mathrm{subject}\;\mathrm{to}\; & \frac{{\left\|x\right\|}_{\infty }^{2}}{\frac{1}{N_{c}N}{\left\|x\right\|}_{2}^{2}}=\gamma \end{array}$$

Let x=tu and ${\left\|u\right\|}_{2}^{2}=1$, the above problem can be rewritten by:

(83) $$\begin{array}{ll} \underset{t,u}{\min} & t^{2}-2tℜ\left\{u^{H}v_{k}\right\}\\ \mathrm{subject}\;\mathrm{to}\; & t>0\\ & {\left|u_{n}\right|}^{2}\leq \frac{\gamma }{lN_{c}N}\\ & {\left\|u\right\|}_{2}^{2}=1 \end{array}$$

where

(84) $$v_{k}=A_{k+1}c_{k+1}+\frac{\lambda_{k}}{\rho }$$

The constraint of t>0; therefore, the variables t and u can be optimized separately. To obtain u, the optimization problem can be formulated by:

(85) $$\begin{array}{ll} \underset{u}{\max} & ℜ\left\{u^{H}v_{k}\right\}\\ \mathrm{subject}\;\mathrm{to}\; & {\left|u_{n}\right|}^{2}\leq \frac{\gamma }{lN_{c}N}\\ & {\left\|u\right\|}_{2}^{2}=1 \end{array}$$

The equality constraint can be relaxed as an inequality constraint. Then the new problem shown below is equivalent to (85) [33].

(86) $$\begin{array}{ll} \underset{u}{\min} & -ℜ\left\{u^{H}v_{k}\right\}\\ \mathrm{subject}\;\mathrm{to}\; & {\left|u_{n}\right|}^{2}\leq \frac{\gamma }{N_{c}N}\\ & {\left\|u\right\|}_{2}^{2}\leq 1 \end{array}$$

The optimization problem (86) is convex. Hence, it is easy to find its solution and the Lagrange function corresponding to (86). The Lagrange function is formulated as:

(87) $$ℰ(u,\mu_{k})=-ℜ\left\{u^{H}v_{k}\right\}+\mu_{k}\left({\left\|u\right\|}_{2}^{2}-1\right)=\sum_{n=1}^{lN_{c}N}ℜ\left\{u_{n}^{*}v_{k,n}\right\}+\mu_{k}\left(\sum_{n=1}^{lN_{c}N}{\left|u_{n}\right|}_{2}^{2}-1\right)$$

The new optimization problem is formulated by:

(88) $$\begin{array}{cc} \underset{u}{\min} & ℰ(u,\mu_{k})\\ \mathrm{subject}\;\mathrm{to}\; & |u_{n}{|}^{2}\leq \frac{\gamma }{N_{c}N} \end{array}$$

The problem (88) can be decomposed into $N_{c}N$ subproblems. Therefore, the *n*-th subproblem can be written by:

(89) $$\begin{array}{cc} \underset{u_{n},\mu_{k}}{\min} & -ℜ\left(u_{n}^{*}v_{k,n}\right)+\mu_{k}{\left|u_{n}\right|}^{2}\\ \;\mathrm{subject}\;\mathrm{to}\; & \left|u_{n}\right|\leq \sqrt{\frac{\gamma }{N_{c}N}} \end{array}$$

and the elements of variable u can be obtained separately by solving (89), the solution can be given by:

(90) $$u_{n}=\{\begin{array}{c} \frac{v_{k,n}}{2\mu_{k}},\;\frac{\left|v_{k,n}\right|}{2\mu_{k}}<\sqrt{\frac{\gamma }{N}}\\ \sqrt{\frac{\alpha }{N}}e^{j\phi \left(v_{k,n}\right)},\;\;\mathrm{otherwise}\; \end{array}$$

Additionally, the dual variable $\mu_{k}$ can be given by using binary section searching method. On account of $u_{n}^{*}v_{k,n}>0$, the variable t can be computed by:

(91) $$t_{t+1}=ℜ\left(u^{H}v_{k}\right)$$

(4) Updating $\lambda_{k+1}$

The dual variable can be updated with

(92) $$\lambda_{k+1}=\lambda_{k}+\rho \left(A_{k+1}c_{k+1}-x_{k+1}\right)$$

**Algorithm 2:** Solving the optimization problem (53) using POADMM**Initialization:** Initialize (s,c,x,λ) as ($s_{0},c_{0},x_{0},\lambda_{0}$) and The iteration index is set to be *k* = 0. Let *K* is the maximum number of iterations. Initialize the thresholds $\epsilon_{1}$,$\epsilon_{2}$ and $\epsilon_{3}$. **For**
*k* = 1,2,3,…*K* Solve the subproblem (61).Calculate the expressions (72) and (73). Then solve the equivalent optimization problem (71) to update ***s****_k+1_* using the POBFGS.Solve the subproblem (62).Calculate the expression (80). Solve the optimization problem (79) to obtain ***c****_k+1_* using the POBFGS.Solve the subproblem (63).Calculate the expressions (90) and (91). Then compute $x_{k+1}=tu$.Solve the subproblem (64) using the expression (92) to update the dual variable.**Until** some preset terminal conditions are satisfied [32]. Then let the optimal waveform be $s_{opt}=s_{k+1}$.

Consequently, the optimal solution can be obtained by the iteration method of solving the expressions (61)–(64) until some stopping criterion is satisfied. For example, the conditions, including ${\left\|A_{k+1}c_{k+1}-x_{k+1}\right\|}_{2}^{}<\epsilon_{1}$, ${\left\|s_{k+1}^{H}\Xi s_{k+1}-s_{k}^{H}\Xi s_{k}\right\|}_{2}<\epsilon_{2}$ and ${\left\|\lambda_{k+1}-\lambda_{k}\right\|}_{2}<\epsilon_{3}$ are satisfied simultaneously and the thresholds $\epsilon_{1}$, $\epsilon_{2}$, and $\epsilon_{3}$ should be given initially. POADMM is illustrated in Algorithm 2. The computational complexity in each iteration is roughly $O\left(N_{s}^{2}\right)$ owing to that the number of iterations of BFGS is much fewer than $N_{s}$ through experiencing many simulations in this paper. The symbol $N_{s}$ is equal to the length of the vector *s* in expression (55). In addition, according to reference [32], the proposed method based on the ADMM converge to a locally optimal point.

## 5. Numerical Simulation

This section provides various necessary numerical simulations to assess the performance of the proposed schemes. It is worth mentioning that the manners of extracting the range information using the OFDM radar and the traditional radar using chirp signals are different. Exactly, the OFDM radar uses the Fourier transform, and the inverse Fourier transform while the chirp-based radar exploits the pulse compressing technique. Then many existing methods cannot be applied to the OFDM radar. Hence, few methods can be used to compare with the proposed method in this paper because the techniques are not universal. However, some useful methods in the open literature are employed to compare with the proposed approaches. In [13], the random initial phases and POCG are used to suppress false targets. Then we compare POCG and the method based on the random phase codes with the proposed schemes. The jamming-to-signal power ratio (JSR) is set to be 40 dB in this section.

### 5.1. The ECCM Performance of Resisting the VDJ

**Figure 4:**
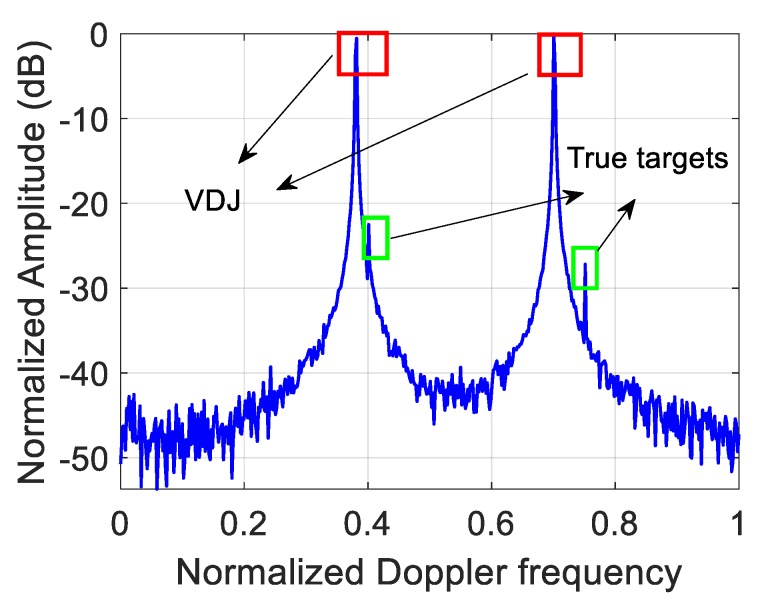
The countermeasure effect in the presence of the VDJ.

For the VDJ, the simulation parameters are listed in Table 1. Not only are the false targets powerful enough to deceive the radar, but their power can almost drown the true ones, which is shown in Figure 4. The deceptive jamming succeeds in dragging false targets in the Doppler domain.

**Figure 5:**
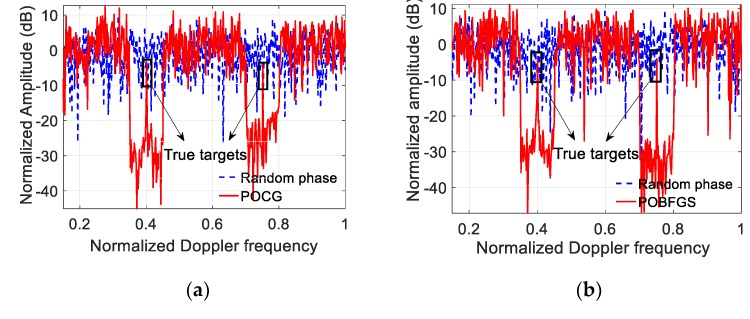
The results of resisting the VDJ using the different methods. (**a**) Comparison of random phase and POCG. (**b**) Comparison of random phase and POCG.

**Figure 6:**
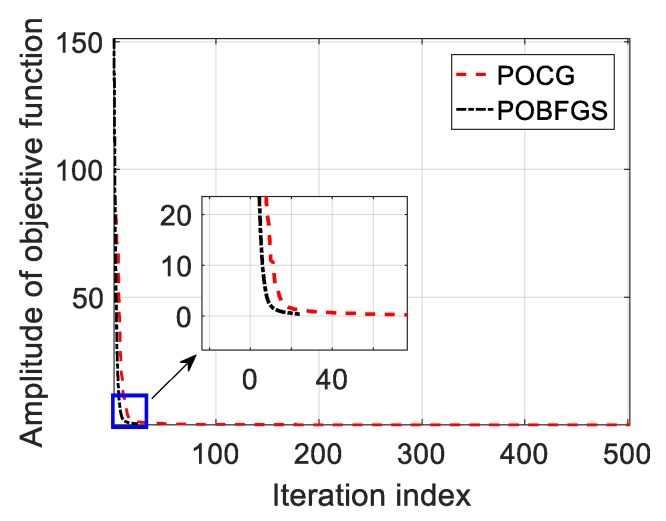
The convergence performance of the methods resisting the VDJ.

In order to highlight the effect of radar ECCM, the ECCM results using the existing POCG and the random coding method are shown in Figure 5a, and the results of both the random coding method and the proposed scheme are compared in Figure 5b. Although the jamming energy cannot be concentrated using these approaches, there are some differences among them. Exactly, in the case of encoding the initial phases of the OFDM pulses by means of the random phase, the true targets are still submerged. However, two notches are formed using POCG and POBFGS since the energy distribution in the specified Doppler intervals near the false targets is subdued. As a result, the energy peaks with regard to the true targets appear. Through statistics, the average SINRs in the two notches obtained by POCG and POBFGS are about 22.66 dB and 26.5 dB, respectively. The performance of suppressing jamming using the POBFGS is better. To assess the performance of the convergence property, we compare three methods, including POCG and the proposed POBFGS in Figure 6. By comparison, the method of POBFGS converges faster than POCG. Under the same terminal conditions, the proposed POBFGS ends first. Hence, the proposed POBFGS is better than the existing methods.

### 5.2. The ECCM Performance of Resisting RDJ and JRVDJ

In the case of resisting RDJ and JRVDJ, because the coded objects are the subcarriers of the relevant OFDM pulses, the coding manner has an impact on the PAPR of the OFDM signal. Then controlling the PAPR must be considered when an anti-jamming operation is implemented. To verify the effectiveness of the proposed method, we build a simulation scenario for the cases of suppressing the RDJ and JRVDJ, respectively. Then the results of the method of random encoding and the proposed schemes are shown in this subsection. Additionally, the parameter of the global PAPR is set to be *γ* = 2 (i.e., 3 dB) in expression (55). Since there are few investigations on the problem proposed in this paper, only the results of our proposed methods are presented.

**Figure 7:**
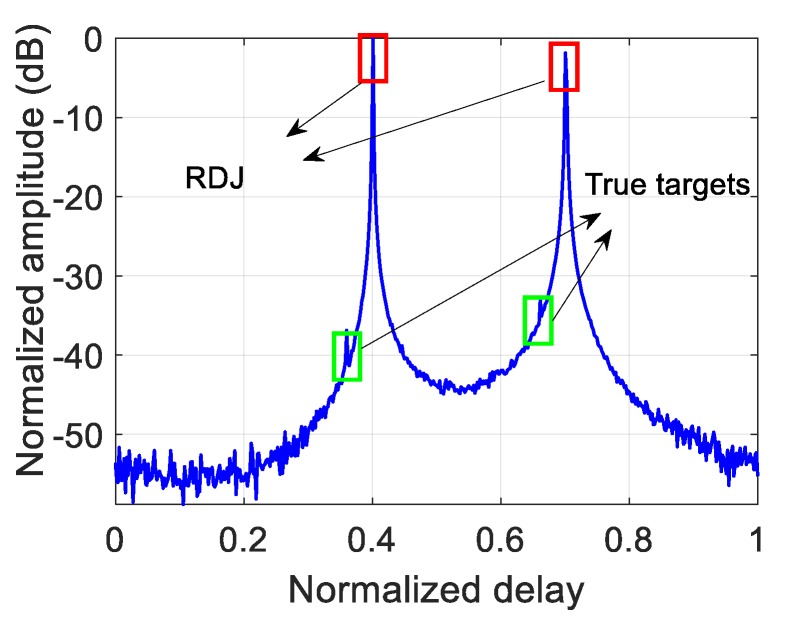
The countermeasure effect in the presence of the RDJ.

**Figure 8:**
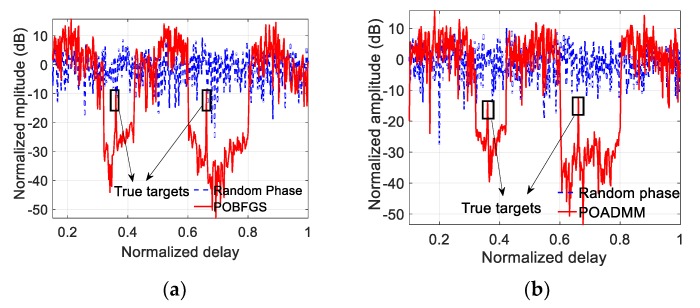
The effect of resisting the RDJ using different methods. (**a**) The result of POBFGS without the constraint of global PAPR. (**b**) The result of POADMM with the constraint of global PAPR.

**Figure 9:**
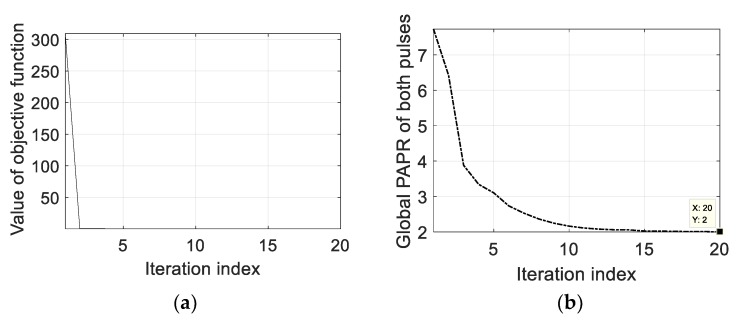
The convergence performance of POADMM and the variation of the global PAPR in the case of suppressing RDJ. (**a**) The convergence performance of the objective function using POADMM in the case of resisting RDJ. (**b**) The variation of the global PAPR in the case of resisting RDJ.

In the case of resisting RDJ, the simulation parameters are shown in Table 2. The countermeasure effect is illustrated in Figure 7, where the true targets can hardly be detected. After optimizing the PCSs, the ECCM effect of using POBFGS without considering the constraint of PAPR is shown in Figure 8a and the result of POADMM is given in Figure 8b with the existence of the constraint of the global PAPR. It can be seen that the true targets can be easily found in the delay notches. The average signal-to-interference-plus-noise ratio (SINR) in the two notches is 24.68 dB in Figure 8a, while the average SINR is 20.81 dB in Figure 8b. The reason why the performance in Figure 8a is better than that in Figure 8b is that the degrees of freedom in the condition of ignoring the PAPR constraint is larger than that constrained by the global PAPR. For the sake of investigating the convergence property of the objective function and the variation of the global PAPR of both OFDM pulses, the relevant results are shown in Figure 9a,b, respectively. The value of the objective function decreases monotonously in light of Figure 9a. In Figure 9b, the global PAPR does not have to be monotonous, but the global PAPR approaches 2.

**Figure 10:**
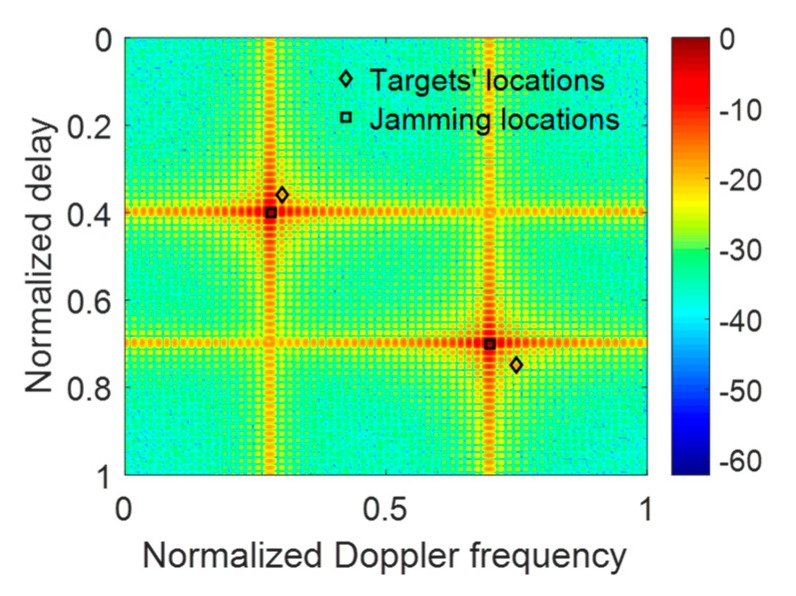
The countermeasure effect in the presence of the JRVDJ.

**Figure 11:**
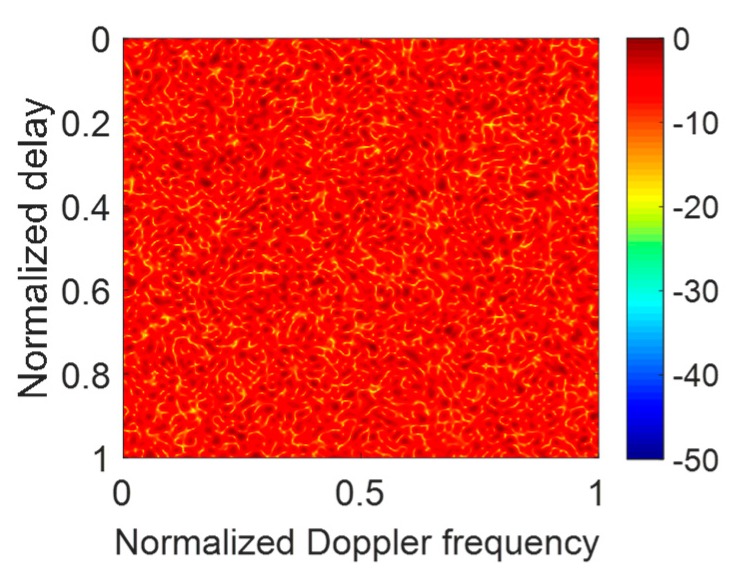
The result of suppressing the JRVDJ using the method of encoding the PCSs with random phase codes.

**Figure 12:**
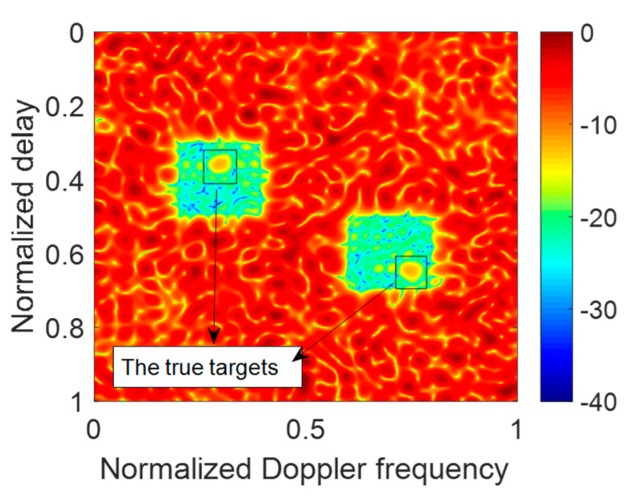
The result of suppressing the JRVDJ using POADMM.

**Figure 13:**
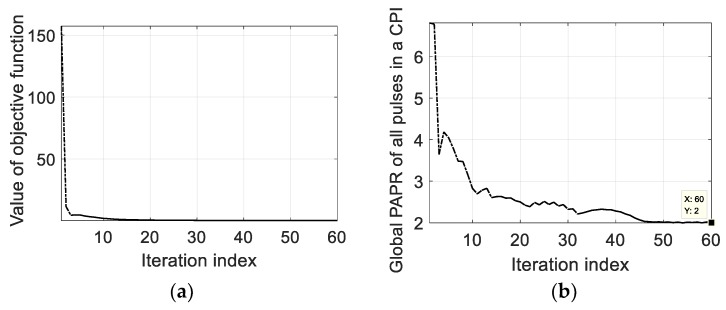
The convergence performance of POADMM and the variation of the global PAPR in the case of suppressing JRVDJ. (**a**) The convergence performance of the objective function using POADMM in the case of resisting JRVDJ. (**b**) The variation of the global PAPR in the case of resisting JRVDJ.

The simulation parameters of resisting the JRVDJ are listed in Table 3, and the expression form of the target or jamming information is denoted by (the normalized delay, the normalized Doppler frequency). For example, the information surrounded by parenthesis of the target, (0.3, 0.36), means that the normalized delay and the normalized Doppler frequency are 0.3 and 0.36, respectively. The effect of dragging the targets in the Range–Doppler domain is simulated in Figure 10. The ECM effect can disable the sensing function of the radar. To disperse the jamming energy of the peaks, the PCSs of all the OFDM pulses in a CPI can be generated randomly. As shown in Figure 11, the level of the energy base rises, which causes the targets to be overwhelmed. For the purpose of controlling the global PAPR with regard to all the OFDM pulses, the effect of our proposed approach is described in Figure 12 where the average SINR is improved to be 16.58 dB in the notches. Moreover, the convergence performance of the objective function and the variation trend of the global PAPR are respectively drawn in Figure 13a,b. According to Figure 13a, the value of the objective function reduces monotonously. Ultimately, as shown in Figure 13b, the global PAPR is approaching to 2 in the end.

## 6. Conclusions

In this paper, a new optimization problem with the constraint of the global PAPR is formulated for the OFDM radar for the first time to suppress the energy of RDJ and JRVDJ in certain areas in order to improve the local SINR. To solve the formulated problem, we develop two methods involving POBFGS and POADMM, which can successfully optimize the OFDM pulses. The anti-jamming effect has been validated in terms of the simulation. The performance of the POBFGS is better than the existing methods in the aspect of resisting VDJ. For the RDJ and JRVDJ, the POADMM succeeds in solving the formulated optimization problem effectively. The algorithm has taken full advantage of the pulse diversity of the OFDM signal for the purpose of ECCM. Motivated by the demands of the future battlefield, the joint of the radar and communication system is becoming one of the most popular topics. It is worth mentioning that the communication function should be considered to be added to the OFDM radar system. Our future direction is to verify the effectiveness of the proposed methods in the practical experimental platform. Another goal of our research is to develop a new method to modify or substitute the POBFGS framework to further improve the computational efficiency of obtaining the initial phase vector to resist VDJ and the solutions to POADMM to suppress RDJ together with JRVDJ.

## Figures and Tables

**Table 1:** Simulation Parameters for Suppressing VDJ.

Physical Meanings	Quantity
The number of the subcarriers	512
The number of the pulses in a CPI	512
Signal-to-noise ratio	0 dB
Jamming-to-signal ratio	40 dB
The normalized Doppler frequency of the targets	0.4, 0.75
The normalized Doppler frequency of the VJs	0.38, 0.72
The suppressed intervals of the normalized Doppler frequency	[0.35, 0.45], [0.7, 0.8]

**Table 2:** Simulation Parameters for Suppressing RDJ.

Physical Meanings	Quantity
The number of the subcarriers	512
The number of the pulses in a CPI	512
Signal-to-noise ratio	0 dB
Jamming-to-signal ratio	40 dB
The normalized delay of the targets	0.36, 0.66
The normalized delay of the RJs	0.4, 0.7
The suppressed intervals of the normalized delay	[0.32, 0.42], [0.6, 0.8]

**Table 3:** Simulation Parameters for Suppressing JRVDJ.

Physical Meanings	Quantity
The number of the targets	2
The number of the subcarriers	512
The number of the pulses in a CPI	512
Signal-to-noise ratio	0 dB
Jamming-to-signal ratio	40 dB
The information of the targets	(0.3,0.36), (0.75,0.65)
The information of the JRVDJ	(0.28,0.4), (0.7,0.6)
The suppressed intervals of the normalized Doppler frequency	[0.2, 0.4], [0.6, 0.8]
The suppressed intervals of the normalized delay	[0.3, 0.5], [0.5, 0.7]

## Appendix A

Detailed Derivation of (73)

By splitting $B_{k}$ into two parts, the objective function of the optimization problem (71) can be expanded to

(A1) $$\begin{array}{ll} {ℱ}_{s}\left(s\right) & =s^{H}\Xi s+\frac{\rho }{2}{\left\|\left[\begin{array}{cc} B_{k,0} & B_{k,\Delta } \end{array}\right]\left[\begin{array}{l} 1_{N}\\ s_{\Delta } \end{array}\right]-x_{k}+\frac{\lambda_{k}}{\rho }\right\|}_{2}^{2}\\ & =s^{H}\Xi s+\frac{\rho }{2}{\left\|B_{k,\Delta }s_{\Delta }+p\right\|}_{2}^{2}\\ & =s^{H}\Xi s+\frac{\rho }{2}\left(s_{\Delta }^{H}B_{k,\Delta }^{H}B_{k,\Delta }s_{\Delta }+s_{\Delta }^{H}B_{k,\Delta }^{H}p+p^{H}B_{k,\Delta }s_{\Delta }+p^{H}p\right) \end{array}$$

where $p=B_{k,0}1_{N}-x_{k}+\lambda_{k}/\rho$.

And $B_{k,0}$ and $B_{k,\Delta }$ are respectively formed by the first *N* columns and left columns of *B_k_*. Concretely, $B_{k,\Delta }$ can be expressed by

$$B_{k,\Delta }=\left(I_{N_{c}}⊗M_{IDFT}\right)\left[\begin{array}{c} 0_{N\times N}\\ I_{N_{c}-1}⊗\mathrm{Diag}(c) \end{array}\right]$$

Because of $M_{IDFT}$ is a unitary matrix, $M_{IDFT}^{H}M_{IDFT}=1$ is obtained. Then $B_{k,\Delta }^{H}B_{k,\Delta }=0_{N_{c}\times N}$. We get $s_{\Delta }^{H}B_{k,\Delta }^{H}B_{k,\Delta }s_{\Delta }=0$.

(A2) $${ℱ}_{s}\left(s\right)=g\left(s\right)+\frac{\rho }{2}r\left(s_{\Delta }\right)+\frac{\rho }{2}\left(N_{p}+{\left\|p\right\|}^{2}\right)$$

where $g\left(s\right)=s^{H}\Xi s$, $q={\left(p^{H}B_{k,\Delta }\right)}^{T}$ and $r\left(s_{\Delta }\right)=s_{\Delta }^{H}B_{k,\Delta }^{H}p+q^{T}s_{\Delta }$. The vector q is not related to ***s***.

## Appendix B

Detailed Derivation of (79)

The objective function in (80) can be expanded to

(A3) $${ℱ}_{c}\left(c\right)=c^{H}\Lambda c-c^{H}A_{k+1}^{H}\hat{p}-{\hat{p}}^{H}A_{k+1}c+{\left\|\hat{p}\right\|}^{2}$$

where $\Lambda =A_{k+1}^{H}A_{k+1}$, $\hat{p}=x_{k}-\lambda_{k}/\rho$. The gradient of ${ℱ}_{c}\left(c\right)$ with respect to $\eta_{c}=\arg\left(c\right)$ can be given by

(A4) $$\begin{array}{ll} \nabla_{\eta_{c}}{ℱ}_{c}\left(c\right) & =\nabla_{\eta_{c}}\kappa \left(c\right)+j\left\{c^{*}⊙\left[A_{k+1}^{H}\hat{p}\right]\right\}-j\left\{{\left[{\hat{p}}^{H}A_{k+1}\right]}^{T}⊙c\right\}\\ & =\nabla_{\eta_{c}}\kappa \left(c\right)+2ℑ\left\{{\left[{\hat{p}}^{H}A_{k+1}\right]}^{T}⊙c\right\} \end{array}$$

where $\nabla_{\eta_{c}}\kappa \left(c\right)=ℑ\left\{diag\left(\Lambda cc^{H}-cc^{H}\Lambda \right)\right\}$.
